# Accelerated discharge of patients in the event of a major incident: observational study of a teaching hospital

**DOI:** 10.1186/1471-2458-6-108

**Published:** 2006-04-26

**Authors:** Kirsty Challen, Darren Walter

**Affiliations:** 1 Emergency Department, South Manchester University Hospitals Trust, Southmoor Road, Manchester M23 9LT, UK

## Abstract

**Background:**

Since October 2002 in the UK Primary Care Trusts (PCTs) have had statutory responsibility for having and maintaining a Major Incident plan and since 2005 they have been obliged to co-operate with other responders to an incident. We aimed to establish the number of beds in our Trust which could be freed up over set periods of time in the event of a major incident and the nature and quantity of support which might be required from PCTs in order to achieve this.

**Methods:**

Repeated survey over 12 days in 3 months of hospital bed occupancy by type of condition and discharge capacity in an 855-bed UK tertiary teaching hospital also providing secondary care services. Outcome measures were bed spaces which could be generated, timescale over which this could happen and level and type of PCT support which would be required to achieve this.

**Results:**

Mean beds available were 78 immediately, a further 69 in 1–4 hours and a further 155 in 4–12 hours, generating a total of 302 beds (36% of hospital capacity) within 12 hours of an incident. This would require support from a PCT of 150,000 population of 10 nursing care beds, 20 therapy-supported intermediate care beds, and 25 care packages in patients' own homes.

**Conclusion:**

In order to fulfill the requirements of the Civil Contingencies Act 2004, PCTs should plan to have surge capacity in the order of 30 residential placements and 25 community support packages per 150,000 population to support Acute Trusts in the event of a major incident.

## Background

Since October 2002 UK Primary Care Trusts (PCTs), the major providers of community health care services, have had statutory responsibility for having and maintaining a Major Incident plan [1]. With the implementation of the Civil Contingencies Act 2004, PCTs have been designated Level 1 responders, with an obligation to co-operate with other Level 1 responders to an incident. Specifically, Department of Health guidance states that primary care organizations will "assist acute trusts by providing staff where appropriate and supporting accelerated discharge" and "co-ordinate community hospital bed capacity in liaison with local acute hospitals and any available local bed management system"[2].

South Manchester University Hospital (Wythenshawe) has 855 beds with onsite orthopaedic, general, vascular, maxillofacial, burns and plastic surgery and cardiothoracic surgery including a transplant unit. The Radiology department operates 2 CT scanners and an MR scanner. Neurosurgical facilities are located at Hope Hospital, Salford, less than 10 miles away. The Trust provides secondary care mostly for patients from South Manchester, Trafford and Stockport Primary Care Trusts with significant numbers also from East Cheshire. The Emergency Department sees around 80,000 new patients/year. Within the Trust's primary catchment area are Manchester International Airport, the M56/M60 interchange, the Metrolink tram system and industrial premises storing petroleum and gas and using cyanide, toluene, acetylene, methane and a variety of biohazardous materials. Within 5 miles is Old Trafford football ground (capacity 70,000) and the Trafford Shopping Centre, which attracts 520,000 shoppers each week[3]. There were 108 major incidents declared in the UK between 1968 and 1996, the largest of which required 90 (Hillsborough), 75 (Bradford stadium fire) and 73 (M1 Kegworth air crash) admissions to hospital[4]. A further 36 incidents affecting health services have been identified between 1997 and 2004, the largest being the Southall train crash (180 casualties), the Omagh bombing (100 casualties) and the July 2001 Bradford riots (80 casualties)[5]. The last major incident seen at Wythenshawe Hospital was the Manchester air crash in 1985, where 76 patients were seen and 15 admitted of which 5 were ventilated[6].

Models are available for the simulation of mass casualty handling in terms of surgical capability[7,8] but these assume no bottleneck or delay from theatre to ward. Internationally, Israeli and Dutch hospitals are required by statute to cope with casualties equivalent to 15–20% and 3% of bed capacity respectively[9,10]. St Vincent's Hospital in Manhattan cleared 50 of 550 beds for casualties on 11^th ^September 2001[11]. No similar data or modelling is currently available for discharge capacity for UK hospitals and we therefore aimed to establish the number of beds in our Trust which could be freed up over a period of time in the event of a major incident and the nature and quantity of support which might be required from Primary Care Trusts in order to achieve this.

## Methods

The whole hospital bed capacity was surveyed over the course of a single working day on 12 different occasions. Surveys were undertaken by one researcher to minimize inter-rater variability. Days surveyed were four each of Monday, Wednesday and Friday (to allow for day-to-day variation in bed occupancy) chosen across three months on a convenience basis. A starting point in the hospital was picked at random to counteract, as far as possible, bias generated by the length of time taken to complete the assessment.

Primary endpoints were: an immediately available bed, discharge feasible within 4 hours, discharge feasible within 4–12 hours and patient still requiring inpatient care. Nursing notes available on the ward (as this reflects what would be available during a major incident) were used to gather data on patient demographics and on the factors contributing to the patient's ongoing stay (eg recent surgery, ongoing intravenous therapy, requiring placement in nursing home). Where nursing notes required clarification further information was sought from nursing staff. Patients were categorized into endpoints using the European amendment of the Appropriateness Evaluation Protocol[12]. Where categorization was not evident from the notes, an endpoint was reached by consensus between local nursing staff and the researchers.

Anonymised data was entered into and analysed using SPSS 11.5 (SPSS inc.). Ethical approval was obtained from Stockport LREC.

## Results

**Figure 1:**
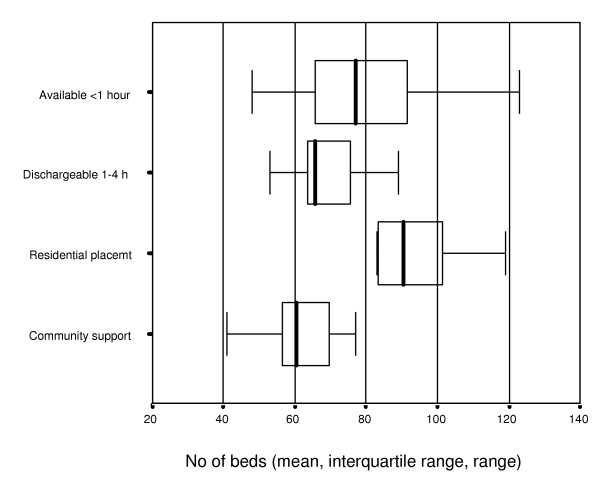
Numbers of available beds.

Numbers of available beds are shown in Fig 1 and table 1. 43% of the patients dischargeable in 1–4 hours are elective or semi-elective preoperative patients whose surgery would be postponed in the event of a major incident and 35% are patients whose discharge that day is already planned. It is assumed that for those patients who require placement in nursing or residential care, or who could be discharged with enhanced community care, this would take in excess of 4 hours to organize. Table 2 shows the age and gender distribution of these groups.

**Figure 2:**
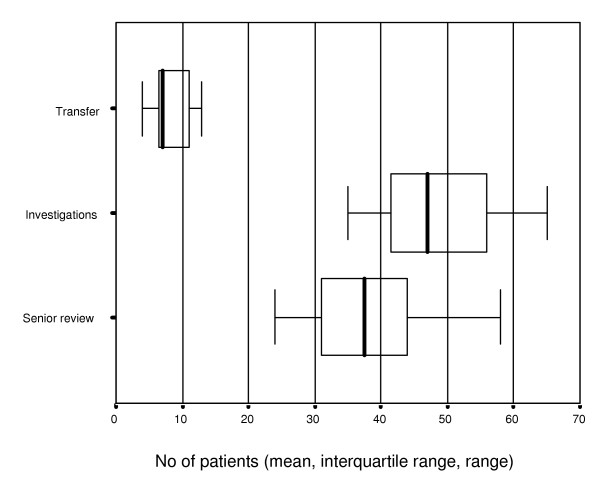
Numbers of potential discharges.

Fig 2 and table 1 show the numbers of patients who could potentially be discharged pending action by the Acute Trust, either by transferring out to other hospitals (usually after specialist treatment, mostly angioplasty), discharging those awaiting, as an inpatient, investigations which could be carried out on an outpatient basis, or by accelerating senior medical review. 59% of patients awaiting investigations were medical, and 7% urological.

A mean of 317 patients (range 249–345) were receiving care for which an inpatient stay was still required.

**Figure 3:**
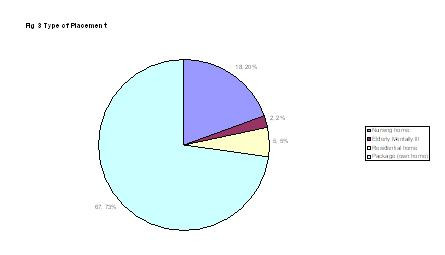
Types of community placement required.

**Figure 4:**
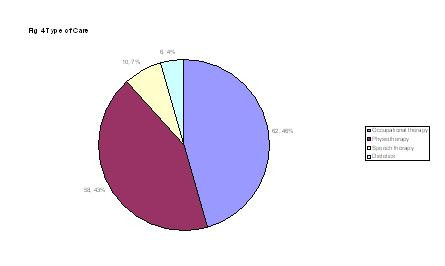
Types of care/therapy required.

Of the patients requiring placement, care packages or enhanced care, figs 3 and 4 show the types of placement and care required:

Table 3 shows the contribution which might be expected from the lead local PCTs.

It should be noted that in the event of a pan-Manchester major incident Trafford and Stockport PCTs would also have to cope with discharges from Trafford General and Stepping Hill Hospital (Stockport) respectively.

## Discussion

We have demonstrated the likelihood that our Trust would, in the event of a major incident, almost immediately be able to create beds to accommodate around 120 patients by cancelling elective surgery, discharging all preoperative patients and by accelerating already planned discharges. A PCT of 150,000 population would need to contribute to this by providing urgent placements for 55 patients, 25 in patients' homes, 10 in nursing or residential care, and 20 in interim therapy-supported beds (of the kind already supported as intermediate care). More potential discharges would undoubtedly be identified by senior medical review of inpatients at the time of the major incident[13].

Given the repeated surveys undertaken of the hospital over varying days and 3 months, it is likely that our findings are representative. It is possible that by using only information documented in the nursing notes (unless this required clarification) we have underestimated the number of patients who would have potential to be discharged in a major incident. However it was clear that personal interaction with a member of staff caring for each patient by a single researcher (to minimise inter-rater variability) was unachievable within a reasonable timescale.

Although there are reports of the bed management achievements in other hospitals during major incidents, none of these relates to the UK and as far as we are aware this is the first scoping exercise aiming to define the potential capacity of an acute Trust and scope the requirement for PCT support pre-incident.

It is not clear how appropriate it would be to generalize our data to other hospitals and primary care organizations, given the presence of particularly deprived electoral wards in our catchment area[14]. It is likely that our Trust is relatively representative of other urban hospitals in deprived areas but generalizing from our data may overestimate the number of beds available in acute Trusts in more affluent areas where social factors are less of an issue in discharge planning. It might therefore be appropriate for this process to be repeated in other acute trusts with different demographics as part of their planning process.

## Conclusion

Given the new responsibilities of Primary Care Organisations, as Level 1 responders under the CCA, to support local acute trusts, our work would suggest that a Primary Care Trust covering a population of 150,000 should plan to be able to accommodate around 50–60 patients (25 in patients' homes, 10 in nursing care and 20 in therapy-supported intermediate beds) as surge capacity in the event of a major incident.

## Competing interests

The author(s) declare that they have no competing interests.

## Authors' contributions

DW and KC jointly conceived and planned the study. KC carried out data collection and analysis. The paper was jointly drafted.

## Pre-publication history

The pre-publication history for this paper can be accessed here:



## Figures and Tables

**Table 1:** 

**Bed category**	**Mean**	**Range**
Available <1 hour	77	48–123
Dischargeable 1–4 hour	66	53–89
Requiring residential care	90	83–119
Requiring community support	60	41–77
Awaiting transfer	6	4–13
Awaiting investigations	46	35–65
Awaiting senior review	37	24–58

**Table 2:** 

**Category**	**Male/female**	**Aged ≥65/<65**
Dischargeable 1–4 hours	298/241	127/255
Requiring residential care	437/683	998/99
Requiring community support	249/370	531/75

**Table 3:** 

Number of patients (mean, range)	South Manchester (pop 150,000)	Trafford (pop 210,000)	Stockport (pop 280,000)
Awaiting placement of which:	37 (30–47)	31 (22–45)	6 (3–12)
*Nursing home*	*7 (3–10)*	*5 (1–11)*	*1 (0–4)*
*Residential home*	*2 (0–5)*	*1 (0–4)*	*0 (0–1)*
*Care package in own home*	*26 (19–38)*	*22 (11–35)*	*4 (2–7)*
*Awaiting equipment*	*2 (1–6)*	*3 (0–6)*	*1 (0–3)*
Potential for community supported discharge	21 (14–27)	17 (7–27)	5 (2–7)
